# Ligation of the middle hepatic vein to increase hypertrophy induction during the ALPPS procedure

**DOI:** 10.1007/s00423-021-02181-1

**Published:** 2021-05-10

**Authors:** F. Dondorf, A. Ali Deeb, A. Bauschke, P. Felgendreff, H. M. Tautenhahn, M. Ardelt, U. Settmacher, F. Rauchfuss

**Affiliations:** 1 Department of General, Visceral and Vascular Surgery, Jena University Hospital, Am Klinikum 1, 07747 Jena, Germany; 2 Research Program “Else Kröner-Forschungskolleg AntiAge”, Jena University Hospital, Jena, Germany

**Keywords:** ALPPS, Middle hepatic vein, Hypertrophy induction

## Abstract

**Purpose:**

Here, we analyse the technical modification of the ALPPS procedure, ligating the middle hepatic vein during the first step of the operation to enhance remnant liver hypertrophy.

**Methods:**

In 20 of 37 ALPPS procedures, the middle hepatic vein was ligated during the first step. Hypertrophy of the functional remnant liver volume was assessed in addition to postoperative courses.

**Results:**

Volumetric analysis showed a significant volume increase, especially for patients with colorectal metastases. Pre-existing liver parenchyma damage (odds ratio = 0.717, *p* = 0.017) and preoperative chemotherapy were found to be significant predictors (odds ratio = 0.803, *p* = 0.045) of higher morbidity and mortality. In addition, a survival benefit for maintenance of middle hepatic vein was shown.

**Conclusion:**

This technical modification of the ALPPS procedure can accentuate future liver remnant volume hypertrophy. The higher morbidity and mortality observed are most likely associated with pre-existing parenchymal damage within this group.

## Introduction

The only chance for long-term tumour-free survival in patients with primary or secondary liver tumours is R0 resection [1]. In advanced tumours, the quality and quantity of the future liver remnant (FLRV) is the landmark for resectability. If the residual liver volume is insufficient, patients can develop small-for-size syndrome (SFSS), which has a high risk of mortality [2, 3]. For “normal” parenchyma, an FLRV of ≥ 25% should be left. A higher FLRV (35–40%) is recommended if chronic liver parenchyma damage is present (e.g., due to chemotherapy-associated liver damage, liver fibrosis or cirrhosis) [4–7].

The ALPPS procedure was developed to increase remnant liver volume, in addition to several preoperative interventions. The advantage is rapid hypertrophy of the remaining liver, but morbidity and mortality rates are high [8]. For example, based on collected data from the international ALPPS Registry, Schadde et al. described a morbidity rate of 28% (Clavien-Dindo ≥ IIIb) and a mortality rate of 9%. Subgroup analysis revealed a worse prognosis for older patients (>60 years) and those with non-colorectal liver tumours [9]. The main cause of early postoperative mortality is liver insufficiency due to SFSS. To lower invasivity and to increase hypertrophy, for which the complete mechanism is not fully understood, several modifications of the ALPPS procedure have already been investigated and published. Along with the partial split and laparoscopic approaches, other modifications have been published, including radiofrequency-assisted liver partition with portal vein ligation (RALPP), laparoscopic microwave ablation and portal vein ligation for staged hepatectomy (LAPS), associating liver tourniquet and portal ligation for stage hepatectomy (ALTPS), and sequential ALTPS [10–12].

Based on known conditioning procedures, including portal vein embolization + hepatic vein deprivation [13–15] and experimental work by Schadde et al. that analysed hepatic conditioning without transection [16], we compared “classical” ALPPS with ALPPS plus middle hepatic vein ligation. In our opinion, simultaneous ligation of the middle hepatic vein (MHV) and the right portal vein during the first step of the ALPPS procedure should be accompanied by a further increase in FLRV hypertrophy.

This work summarises our experience with ligation of the MHV during the first step of the ALPPS procedure in view of the hypertrophic increase in the FLRV.

## Methods

Between June 2014 and June 2019, 37 ALPPS procedures with complete parenchyma transection were performed at our centre, similar to the original description of the ALPPS procedure. In 20 of the 37 patients, ligation of the middle hepatic vein (MHV) was performed during the first step. There was no randomisation of the patients, and the choice of the selected operative approach depended on the preference of the surgeon.

The mean age was 64 ± 10.5 years, and the median age of the patients who underwent surgery was 65 years (range 34–78). The indications for surgery and the basic data of the patients are listed in Tables 1, 2 and 3.

**Table 1:** Main characteristics of the two groups: data shown the mean ± standard deviation. Abbreviations: metastases of colorectal carcinoma (CRC), hepatocellular carcinoma (HCC), intrahepatic cholangiocellular carcinoma (CCC). Other: 1 case of adrenal carcinoma metastasis and 1 case of renal cell carcinoma metastasis

		MHV preserved	MHV ligated
		*n* = 17	*n* = 20
Sex	Male	8	9
	Female	9	11
Entity	CRC	10	10
	HCC	3	4
	CCC	3	5
	Other	1	1
Age (a)	Mean	63.5 ± 10	64.4 ± 11.1
BMI (kg/m^2^)	Mean	25.12 ± 4.16	25.36 ± 4.13

**Table 2:** Main characteristics of the tumour entities. Abbreviations: chemotherapy (Cx), metastases of colorectal carcinoma (CRC), hepatocellular carcinoma (HCC), intrahepatic cholangiocellular carcinoma (CCC). Other: 1 case of adrenal carcinoma metastasis and 1 case of renal cell carcinoma metastasis

	Total	Previous surgery	Previous Cx	Period Cx	Time interval Cx/surgery
	*n*	*n* (%)	*n* (%)	month	days
HCC	6	0	0	/	/
CCC	9	0	2 (22.2)	4.5 ± 0.5	/
CRC	20	20 (100)	13 (65)	3.34 ± 0.94	106.5 ± 83.04
Other	2	1 (50)	1 (50)	2	34

**Table 3:** Main characteristics of the two groups. Abbreviation: chemotherapy (Cx)

	Total	Previous surgery	Previous Cx	Period Cx	Time interval Cx/surgery
	*n*	*n* (%)	*n* (%)	month	days
MHV preserved	17	11 (64.7)	8 (47.1)	3.34 ± 0.94	102.8 ± 90.52
MHV ligated	20	10 (50)	8 (40)	5.33 ± 3.2	77.4 ± 50.83

In addition to laboratory chemistry, the LiMAx test (Humedics, Berlin, Germany) was performed prior to step 1 and prior to step 2 (Table 4). All patients were examined by contrast-enhanced CT scans preoperatively, postoperatively immediately after the 1st step, and 1 day before the 2nd step.

**Table 4:** Main results of LiMAx test divided by group—MHV preserved, MHV ligated and divided due to tumour entities. Abbreviation: small-for-size syndrome (SFSS)

	Total	LiMAx prior to step 1	LiMAx prior to step 2	SFSS
	*n*	μg/h/kg	μg/h/kg	*n* (%)
MHV preserved	17	488.77 ± 113.7	430.36 ± 126.33	1 (5.88)
MHV ligated	20	475.27 ± 152.74	437.5 ± 141.43	6 (30)
HCC	6	412 ± 85.94	343.5 ± 83.70	2 (33.3)
CCC	9	513.63 ± 182.14	449.33 ± 175.15	1 (11.1)
CRC	20	483.8 ± 90.7	459.34 ± 120.63	4 (20)
other	2	/	/	0

Subsequently, three-dimensional volumetric analyses were performed with the program Synapse 3D (FUJIFILM, Tokyo, Japan). This software offers the possibility of measuring different liver volumes and calculating tumour volumes.

For volumetric analysis, contrast-enhanced CT is required to which the software can refer. The optimal slice thickness is 0.63 mm.

By extracting the liver in several individual slices (3 axes: axial, sagittal and coronary), the software can generate a three-dimensional reconstruction with (automatic) measurement of total liver volumes. In addition to the future liver remnant volume (FLRV), the total functional liver volume was determined. This was generated by subtracting tumour volumes, tumour volumes < 50 ml were not analysed.

Pre-existing liver parenchyma damage was not detected based on laboratory data, only histologically proven fibrosis of any degree or cirrhosis in the tumour-free liver parenchyma was analysed [17, 18].

As there was no severe steatosis hepatis (>66% of liver parenchyma) in our patient population, steatosis was not considered as parenchyma damage in this analysis.

In this work, the definition of postoperative liver failure in the sense of an SFSS was generated from a combination of different common definitions [3, 19]. The following parameters were combined to assess functional impairment after the second ALPPS step:

- Persistent hyperbilirubinemia with a peak of > 120 μmol/l from the 5th postoperative day. Any biliary complications were excluded AND
- Deviation from normal coagulation status (quick < 65%) despite supportive therapy (vitamin K substitution)

Retrospective collection of basic data was carried out using the program Microsoft Excel Office (Microsoft Cooperation, Redmond, WA, USA). Statistical processing of patient data was performed using the IBM SPSS Statistics 25 (IBM, Armonk, NY, USA) statistical program.

Significance for two independent samples was assessed using the Mann-Whitney *U*-test and we used the Kolmogorov-Smirnov test to check for a normal distribution. The Wilcoxon test was used to observe differences between two paired samples and the z-standardised test was applied to test for significance. The bilateral significance level was 0.05 ± 1.96.

## Results

**Fig. 1:**
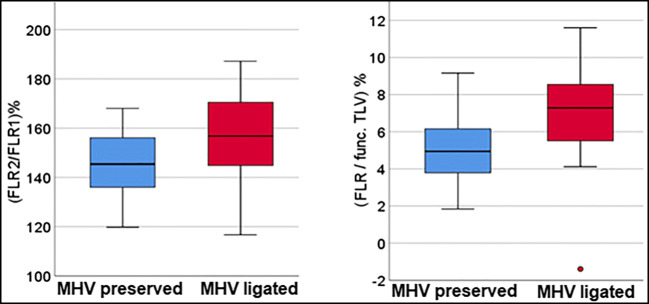
Comparison of the increase in FLR in relation to the two groups: MHV preserved and MHV ligated. Left: Comparison of the ratio (FLRV2/FLRV1) in %. Right: Comparison of the ratio (FLR/total functional liver volume) in %. The total functional liver volume was generated by subtracting the tumour volumes, and tumour volumes <50 ml were not scored

**Fig. 2:**
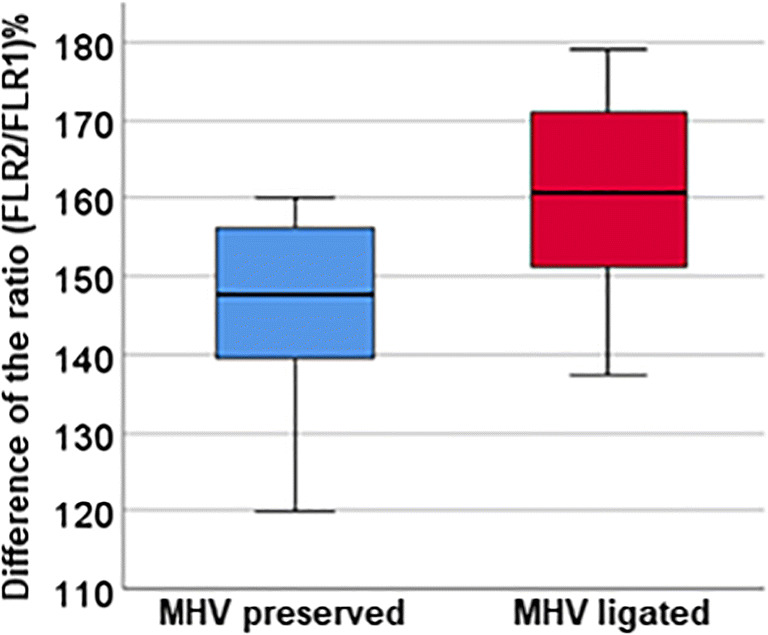
Representation of the ratio (FLRV2/FLRV1) in % in subgroup analysis of CRC patients (*p* = 0.028)

The median increase in FLRV volume in the whole group was 54.1% (range 16.7–96.1%), with a median time interval of 9 days. Subdividing the cohort by the handling of the middle liver vein, an advantage of the MHV ligation group (54.9%, range 16.7–96.1%, in 8.5 days) compared to the conventional ALPPS procedure (49.9%, range 19.8–74.3%, in 10 days) was evident, but not statistically significant (*p* = 0.082). If only the subgroup of patients with colorectal liver metastases was considered, the FLRV2/FLRV1 ratio was statistically significant in favour of MHV ligation (*p* = 0.028) (Table 5, Figs. 1 and 2).

**Table 5:** Main results of volumetries, divided in both groups—MHV preserved, MHV ligated. Abbreviations: functional total liver volume (func. TLV), total body weight (TBW)

	MHV preserved	MHV ligated
FLRV-Increase (ml)	218 (134–333)	221 (70–428)
Percentage of FLRV-Increase (%)	49.9% (19.8–74.3%)	54.9% (16.7–96.1%)
FLRV-Increase/func. TLV (%)	5.54% (1.5–15.4%)	6% (−1.4–15.4%)
FLRV-Increase/TBW (%)	0.29% (0.18–0.46%)	0.3% (0.12–0.64%)

**Fig. 3:**
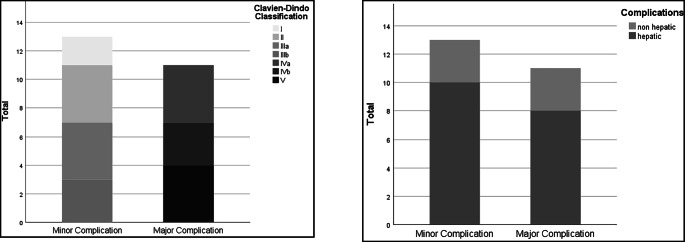
Division of complications into minor and major complications: on the left as division by Clavien-Dindo; on the right by hepatic and non-hepatic complications

**Fig. 4:**
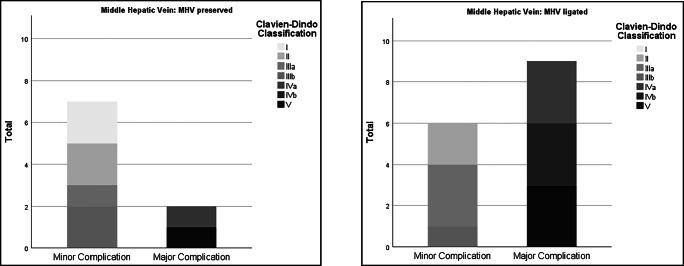
Division of complications into minor and major complications: on the left MHV preserved; on the right MHV ligated

Major complications (Clavien-Dindo > IIIb) occurred in 11 patients (29.7%). Eight patients developed major hepatic complications, of which small for size syndrome was the most common, in 7 patients. One patient experienced prolonged biliary leakage. The remaining three patients had major non-hepatic complications (two pulmonary artery embolisms and one media infarction) (Figs. 3 and 4). Small for size syndrome was the unique cause of 90-day mortality (*n* = 4; 10.8%) (Table 6).

**Table 6:** Presentation of morbidity and mortality after MHV preservation/ligation

	Total	MHV preserved	MHV ligated
	*n* = 37 (%)	*n* = 17 (%)	*n* = 20 (%)
Morbidity (total)	24 (64.9)	9 (52.9)	15 (75)
Major complication	11 (29.7)	2 (11.8)	9 (45)
hepatic complication	18 (48.6)	6 (35.3)	12 (60)
SFSS	7 (18.9)	1 (5.9)	6 (30)
90-day mortality	4 (10.8)	1 (5.9)	3 (15)

As risk factors for postoperative complications and mortality, two major factors were analysed in our cohort. In addition to pre-existing liver parenchyma changes (*p* = 0.017, postoperatively histologically proven fibrosis or cirrhosis), preoperative chemotherapy (*p* = 0.045) was a significant predictor, especially for the development of SFSS.

According to liver parenchyma damage, eight patients in our cohort had mild fibrosis (F1) and three patients had cirrhosis (F4). The remaining 26 patients exhibited no relevant parenchymal changes. Regarding chemotherapy, 59.46% of the patients in our cohort were not treated with preoperative chemotherapy; 24.32% were given folinic acid-based chemotherapy, and the remaining 16.22% had an individual chemotherapy plan.

**Fig. 5:**
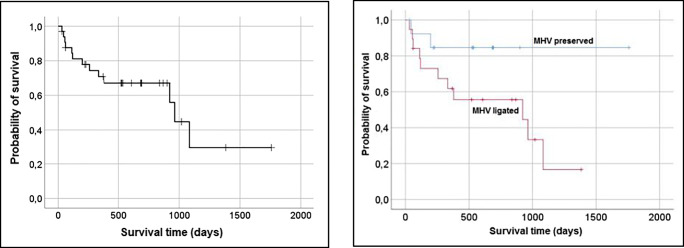
Kaplan-Meier curves showing cumulative overall survival. Left: Overall collective, right: overall collective differentiation by MHV preservation and MHV ligation

Subgroup analysis of the data showed higher morbidity in the MHV ligation group. Overall survival in our cohort was a median survival time of 32 months, and the mean survival time was 31 months (Fig. 5, Tables 7 and 8). Four patients were excluded from the evaluation due to a lack of follow-up information.

**Table 7:** Overall survival (OS) and disease-free survival (DFS) divided by MHV preservation/ligation

		Total	MHV preserved	MHV ligated
		%	%	%
OS	1 year	73.1	90	62.5
	2 years	56.3	50	58.3
DFS	1 year	50	60	42.9
	2 years	14.3	0	29

**Table 8:** Hospital stay, adjuvant therapy, OS, DFS and in-house mortality divided by MHV preservation/ligation

	Total	Hospital stay	Adjuvant therapy	OS	DFS	In house mortality
	*n*	days	*n* (%)	days	days	*n* (%)
MHV preserved	17	35.76 ± 9.66	6 (35.3)	551.43 ± 424.43	325.64 ± 226.56	1 (5.88)
MHV ligated	20	45.6 ± 27.43	6 (30)	525.21 ± 413.77	312.44 ± 342.00	3 (15)

## Discussion

Based on the history and development of the ALPSS procedure, in the very first patient in whom this procedure was performed intraoperatively (emergency) and without prior planning, the middle hepatic vein was divided during the first step [20]. Considering the progression of its development, it must be noted that in the initial description of the procedure, the middle vein was preserved during the first step and was divided during the second step [8]. Thus, routine transection of the middle hepatic vein is not in accordance with the protocol of the initial ALPPS description.

In our opinion, this is the first time that the effect of ligating the MHV in step 1 of the ALPPS procedure has been described in a patient series.

We assumed that in addition to portal vein ligation and complete transection of the parenchyma in step 1, MHV ligation should have an additive effect on hypertrophy induction in the residual liver. The approach of combined interventional venous and portal venous embolization is currently being investigated [21] and appears to support our hypothesis, though the combination of ALPPS and venous occlusion has not yet been described in this way.

In this study, we were only able to identify this effect as statistically significant for CRC-MTS patients in our cohort. Technically, central ligation of the MHV is only required for oncosurgical reasons in patients with tumour load in segment IVa.

In the original description of the procedure, the maintenance of the MHV in step 1 is propagated [8]. Shortly afterwards, the procedure was modified with ligation of the MHV [22]. Performing this interventionally to induce hypertrophy has already been suggested, but for the right liver vein [23]. There are two different current recommendations: supporters of principle ligation [24] and those who in principle receive the MHV in step 1 [25]. However, there is no comparison to date.

The processing of our data revealed completion with the second step after an average of 9 days, though it must be noted that an average of 9–14 days is reported in the literature [26]. Our cohort showed an average time advantage for the MHV ligation group of 8.5 days. This can be explained by faster hypertrophy, even though randomisation was not performed for this study because of the retrospective design.

Hepatic vein embolization induces liver hypertrophy through an increase in portal pressure by regurgitation into the portal vein through the sinusoids [27]. In contrast, the pathophysiology of hypertrophy in ALPPS is still not fully understood, yet similar mechanisms must be present [28]. The combination of both mechanisms was addressed in this technical modification. Additional hypertrophy of the FLRV was shown, albeit not significant in the overall cohort but rather in relation to those with colorectal liver metastases.

Considering that patients with colorectal cancer can benefit from an ALPPS procedure and that the modification with respect to hypertrophy was significant in this patient group, it is obvious that patient selection is of enormous importance, which has already been demonstrated by other research groups [29–31]. In this report, it became also apparent that CRC-MTS patients especially benefit from the ALPPS procedure. In addition to selection of the tumour entity, the liver parenchyma and any pretreatment play a relevant role [25, 32].

Overall, technical modification with ligation of the middle hepatic vein has a positive effect on these patients and can accentuate hypertrophy.

Our analysis showed that pretreatment with chemotherapy and liver parenchyma damage significantly reduce hypertrophy. Nevertheless, further studies are needed, particularly regarding liver regeneration within the setting of ALPPS after chemotherapy [33, 34].

In general, not every patient can be treated by the ALPPS procedure, and further work, including preoperative functional analysis, is necessary [25].

The higher morbidity and mortality of the MHV group can be explained by the retrospective data analysis as a limitation, as the selection of patients was “intuitive” and not randomised intraoperatively; indeed, to achieve a more substantial increase in hypertrophy, patients with a worse parenchymal condition comprised the ligation group. Ultimately, the population reported herein reflects the indication common in the literature. Because the data were collected from 2014 to 2019, it is also possible to observed a change in patient selection towards colorectal metastasis, which was also found by Chan et al. [26].

The patient population presented here had a major complication rate (CD > 3b) of 29.7%. The SFSS rate was 18.9%, and the 90-day mortality rate was 10.8%. Considering this within the context of the literature, several papers show similar results. For example, Vicente et al. found an SFSS rate of 22.2% [35], and Zhang et al. indicated that 75% of ALPPS-associated mortality was due to post hepatectomy liver failure [36]. In addition, a recent review by Chan et al. from 2020 highlights that the main complications of ALPPS are high morbidity (Clavien-Dindo ≥ Grade IIIB complications) and 90-day mortality. Morbidity rates range from 14–50%; however, the incidence of all postoperative complications has been reported to range from 53–90%, with 90-day mortality rates of 0–28.7% [26].

Nevertheless, it must be clearly stated that the decision to ligate MHV was an intraoperative decision by the surgeon and must therefore be mentioned in addition to the lack of randomisation as a limitation. In our opinion, this can be regarded as a technical error in the work and should therefore be further evaluated randomly in additional work; this must be evaluated as a clear selection bias of the study.

## Conclusion

In summary, this technical modification involving ligation of the median hepatic vein during the first step of the ALPPS procedure has a positive influence on the increase in FLRV, but without significance in our series. Further work on ALPPS, subsequent liver regeneration and patient selection is absolutely necessary to minimise the high mortality and morbidity rates and to promote patient safety.
